# The impact of the COVID‐19 pandemic on referral numbers, diagnostic mix, and symptom severity in Eating Disorder Early Intervention Services in England

**DOI:** 10.1002/eat.23836

**Published:** 2022-10-21

**Authors:** Lucy Hyam, Katie L. Richards, Karina L. Allen, Ulrike Schmidt

**Affiliations:** ^1^ Department of Psychological Medicine, King's College London Institute of Psychiatry, Psychology and Neuroscience London UK; ^2^ Centre of Implementation Science, King's College London, Health Service and Population Research Department Institute of Psychiatry, Psychology and Neuroscience London UK; ^3^ Department of Psychological Medicine, King's College London Institute of Psychiatry, Psychology and Neuroscience, London, UK and Eating Disorders Outpatient Service, South London and Maudsley NHS Foundation Trust London UK

**Keywords:** COVID‐19, early intervention, eating disorder services, feeding and eating disorders, national health services

## Abstract

**Objective:**

First Episode Rapid Early Intervention for Eating Disorders (FREED) is a service model and care pathway which aims to provide timely, well‐coordinated, developmentally informed and evidence‐based care for young people with eating disorders (EDs). This article investigates the impact of the COVID‐19 pandemic on FREED patient presentations and service provision in England.

**Method:**

Data from three services spanning the pre‐ to post‐pandemic period were included (January 2019–September 2021; *n* = 502 patients). Run charts were created to analyze changes in monthly baseline patient data (e.g., referral numbers, duration of an untreated ED, diagnostic mix, and average body mass index for patients with anorexia nervosa [AN]).

**Results:**

Significant increases in referral numbers were found from September 2020 onward, coinciding with the end of the first UK national lockdown. The percentage of AN presentations significantly increased after the onset of the first national lockdown (April 2020–December 2020). No other significant change patterns were identified.

**Discussion:**

There have been substantial increases in referral numbers and presentations of AN to FREED services whereas illness severity seems largely unchanged. Together, this suggests that increased referrals cannot be attributed to milder presentations being seen. Implications for the implementation, funding, and sustainability of the model are discussed.

**Public Significance:**

Our research suggests that early intervention eating disorder services across England faced significant increases in patient referrals and presentations of anorexia nervosa over the COVID‐19 pandemic. This increase in referrals is not due to a rise in milder eating disorder cases, as baseline symptom severity remained stable across the pandemic. Investment in early intervention for eating disorders must therefore match increased referral trends.

## 1 INTRODUCTION

Eating disorders (EDs) are common, disabling, and deadly psychiatric illnesses (Treasure et al., 2020). The COVID‐19 pandemic has highlighted the burden of these disorders on individuals, families, and society at large. Evidence suggests that since the start of the pandemic, increases have occurred in the rate of EDs in the community, ED presentations to health services and related hospitalizations, and the acuity and complexity of ED presentations (Haripersad et al., 2021; Shaw et al., 2021; Taquet et al., 2021; Weissman & Hay, 2022). These changes have been observed globally and have led to a call for action on policy, practice, and research relating to EDs (Zipfel et al., 2022).

The median age of onset of EDs is age 18. The 25th to 75th percentiles of onset range from the mid‐teens to mid‐20 s, that is, adolescence to emerging adulthood (Solmi et al., 2022). Young people in this age group are thought to have been particularly adversely affected by the pandemic, given the importance of this period for social, educational, and identity development (Potterton et al., 2021). Governmental infection control measures and the resulting socioeconomic fall‐out (e.g., lockdowns, school closures, job losses) have had major implications for young people (McGorry, 2020; Stroud & Gutman, 2021).

First Episode Rapid Early Intervention for EDs (FREED) is a service model and care pathway for young people (aged 16–25) with recent onset EDs (≤3 years duration). Details on the FREED model, rationale and underpinning evidence can be found elsewhere (e.g., Allen et al., 2020; Austin, Flynn, Richards, et al., 2021; Austin, Flynn, Shearer, et al., 2021; Brown et al., 2018; Flynn et al., 2021; McClelland et al., 2018; Schmidt et al., 2016). Briefly, FREED provides rapid access to well‐coordinated, person‐centered, evidence‐based care that is developmentally informed and tailored to the young person's needs. FREED aims to reduce the duration of an untreated ED to facilitate early recovery and minimize the likelihood of illness progression and chronicity.

In early 2020, FREED was adopted by the Academic Health Science Network (AHSN) for rapid national scaling in England. Data from this rollout up to September 2021 (covering 30 FREED services and 2473 patients) suggest that the model is replicating at scale (Richards et al., 2022). Most of the scaling of FREED has occurred during the COVID‐19 pandemic. Yet, to date, there has been no evaluation of the specific impact of COVID‐19 on FREED patients and service delivery. Systematic reviews synthesizing the evidence on the impact of COVID‐19 on EDs have found mainly negative and some positive influences of the pandemic on ED symptoms in patient populations (Devoe et al., 2022; Schneider et al., 2022). An understanding of the impact on new ED cases and the severity of illness in early stage presentations would be a valuable addition to this literature (Devoe et al., 2022).

The aim of this article is to investigate the impact of the COVID‐19 pandemic on FREED patients and service provision in England.

## 2 METHODS

### 2.1 Design and sample

A repeated cross‐sectional design was utilized to evaluate routinely collected baseline data of FREED patients referred before, and during the COVID‐19 pandemic. The data included here are part of the “FREED‐4‐All” national data set and span from January 2019 to September 2021. For further information regarding the FREED data collection procedures, see Richards et al. (2022). FREED‐4‐All data are not subject to informed consent, which is outlined in an operational agreement signed by participating services. Patients are given the opportunity to opt out of data sharing via a privacy notice and information sheet. Regarding demographic information, the age of participants is collected at referral by clinicians. Data on ethnicity and gender of the sample are not available, as the operational agreement allowing data sharing from FREED services requires data sets to be deidentified with protected characteristics excluded.

During the study period, 30 sites had commenced or newly joined implementation of FREED and contributed data for a total of 2319 FREED patients (16–25 years old, diagnosable ED and duration of an untreated ED ≤3 three years). Only 3 of these 30 FREED sites had data preceding and during the pandemic and could therefore be included here. The sample for the current study included 502 FREED patients from these three FREED services. Sites 1 and 2 have data beginning January 2019, and Site 3 has data beginning February 2020. Patients screened as eligible for FREED are retained in the data set even if they do not have an assessment or start treatment (i.e., the data set includes data on patients who disengage or transfer care).

### 2.2 Outcomes and analysis

The outcomes of interest across all sites included the total number of FREED referrals each month, the mean duration of an untreated ED (in months) of referrals each month, and the percentage of referrals each month that were subsequently diagnosed with AN (excluding atypical AN, compared to all other diagnoses combined). Mean scores on the ED Examination Questionnaire (EDE‐Q; Fairburn & Beglin, 2008) and the Clinical Outcomes in Routine Evaluation‐10/Outcome Measure (CORE‐10/OM; Barkham et al., 2013) were used to assess baseline ED and general psychopathology. For AN patients only, the mean monthly baseline body mass index (BMI; kg/m^2^) was assessed.

Run charts were used to evaluate patterns and variation in each measure over time. Run charts are line graphs that display a measure over time with the median plotted as a horizontal line dividing the data in two, where probability‐based rules objectively determine if patterns in the data are random (i.e., randomly distributed around the median) or nonrandom (i.e., the likelihood of the pattern occurring by chance is *p* < .05) (Anhøj & Olesen, 2014; Perla et al., 2010). In the current study, the data were plotted monthly and two probability‐based rules were applied: *shift* and *run* test rules (outlined in Supplement 1). We analyzed data over a 33‐month period (January 2019–September 2021) relative to the COVID‐19 restrictions in England, as defined by the UK government (Brown & Kirk‐Wade, 2021). Periods included pre‐pandemic (January 2019–February 2020) and reducing restrictions (April 2021–September 2021); national lockdowns (March 2020–June 2020; November 2020; January 2021–March 2021); and minimal, local, or tiered restrictions (July 2020–October 2020; December 2020).

## 3 RESULTS

Table 1 presents the baseline demographic and clinical characteristics of the 502 patients included. There were 149 patients referred before the onset of the first UK COVID‐19 lockdown (March 23, 2020).

**TABLE 1:** Characteristics of FREED patients at baseline over all time points 2019–2021, mean and standard deviation: Mean (*SD*), median (minimum, maximum)

Site		Patients (*n*)	
**1**		**100**	
**2**		**348**	
**3**		**54**	
		***N* = 502**	
**Site**		**Age at referral (years):**	
		**Mean *(SD)***	**Median (min, max)**
1		18.9 (2.22)	19 (16, 25)
2		20.04 (2.39)	19 (17, 25)
3		20.02 (2.04)	20 (17, 25)
**All**		**19.81 (2.36)**	**19 (16, 25)**
		***n* = 501**	
**Site**		**Duration of untreated eating disorder (months):**	
		**Mean *(SD)***	**Median (min, max)**
1		14.01 (9.44)	12 (1, 36)
2		15.09 (9.08)	13 (0, 38)
3		12.43 (10.45)	9 (2, 37)
**All**		**14.42 (9.38)**	**12 (0, 38)**
		***n* = 295**	
**Site**		**Diagnosis: % (*n*)**	
**All**	Anorexia nervosa	42% (194)	
	Bulimia nervosa	17% (77)	
	Binge eating disorder	5% (22)	
	Avoidant/restrictive food intake disorder	1% (6)	
	Other specified feeding or eating disorder	35% (163)	
		***n* = 462**	
**Site**		**BMI for AN patients only:**	
		**Mean *(SD)***	**Median (min, max)**
1		17.78 (2.46)	17.58 (13.44, 25.81)
2		16.78 (1.59)	16.88 (12.40, 19.56)
3		17.64 (1.59)	17.34 (14.33, 23.05)
**All**		**17.47 (2.19)**	**17.35 (12.40, 25.81)**
		***n* = 138**	
**Site**		**Data missingness: % (*n*)**	
**All**	Duration of an untreated eating disorder	41% (207)	
	BMI	11% (56)	
	Diagnosis	8% (40)	
	EDE‐Q	54% (269)	
	CORE‐10/OM	57% (286)	

*Note*: Bold has been used on totals and headings to make the table easier to read visually.

Abbreviations: AN, Anorexia nervosa; BMI, Body Mass Index (BMI; kg/m^2^); CORE‐10/OM, Clinical Outcomes in Routine Evaluation‐10/Outcome Measure; EDE‐Q, Eating Disorder Examination Questionnaire; FREED, First Episode Rapid Early Intervention for Eating Disorders.

**FIGURE 1:**
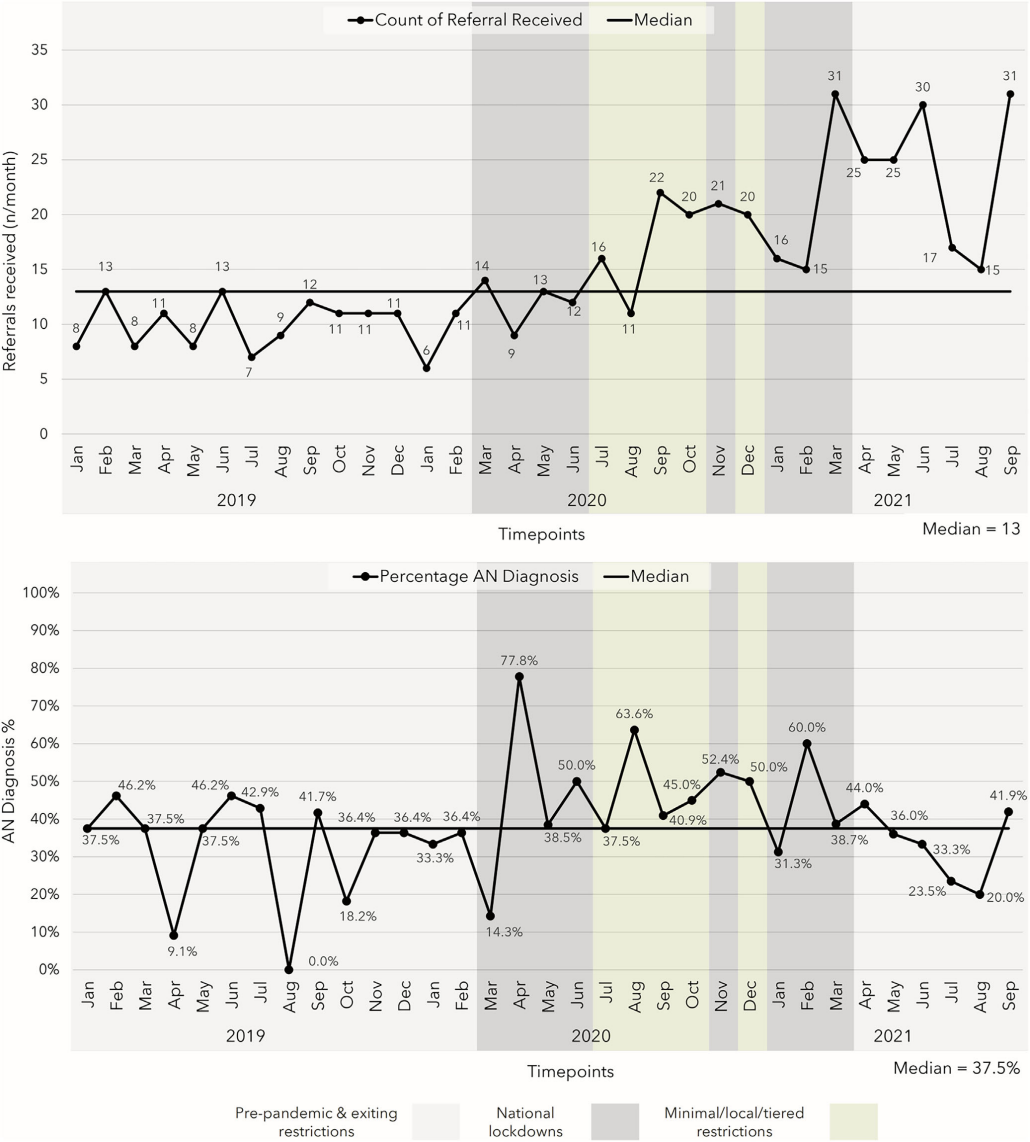
Monthly referral numbers and diagnostic mix (percentage of anorexia nervosa [AN] diagnoses) for First Episode Rapid Early Intervention for Eating Disorders (FREED) patients January 2019–September 2021. The shaded areas of the charts represent the periods of different COVID‐19 pandemic restrictions. Anorexia nervosa (AN) diagnoses exclude atypical AN.

Run charts for referral numbers and diagnostic mix are depicted in Figure 1. Run charts for the monthly average duration of an untreated ED, BMI (for AN only), EDE‐Q global scores, and CORE‐10/OM global scores are in Supplement 2.

### 3.1 Referral numbers

Figure 1 shows that referrals shifted below the median in the period directly preceding the first national lockdown. An upward shift occurred for the period September 2020–September 2021. There were also “too few” runs across the whole time period according to the table of critical values provided in Perla et al., 2010; total referrals fluctuated from below to above the median at a rate which is less than that expected with randomly fluctuating data. These point to an increase in monthly referrals after the first national lockdown that remained consistent until September 2021.

### 3.2 Diagnostic mix

There was a downward shift in the percentage of AN diagnoses relative to other diagnoses for the period directly preceding the pandemic, from October 2019 to March 2020 (Figure 1). The percentage then increased to a peak in April 2020, following the onset of the first national lockdown, and an upward shift emerged until December 2020, suggesting a significant increase in presentations of AN to FREED services during this period.

### 3.3 Duration of an untreated ED

Duration of an untreated ED (in months) across sites showed no indication of nonrandom variation (see Figure S1, Supplement 2).

### 3.4 Body mass index

Figure S1 (Supplement 2) showed an upward shift in mean monthly BMI from February 2019 to September 2019. There were no significant variation patterns that appeared to be related to the onset of COVID‐19 restrictions.

### 3.5 EDE‐Q and CORE‐10/OM

No significant changes were identified for baseline mean global EDE‐Q scores or CORE‐10/OM scores (see Figure S2 Supplement 2).

## 4 DISCUSSION

This study aimed to assess the impact of the COVID‐19 pandemic on FREED patients and service provision. Our main findings are that, first, there was a consistent and substantial increase (~50%) in referral numbers to the FREED services included here from the end of the first national lockdown onward. Second, presentations of AN diagnoses increased at the onset of the first national lockdown until December 2020. Duration of an untreated ED and symptom severity stayed relatively stable across the pandemic.

Our study is limited by the use of flawed, uncertain, proximate and sparse data, ubiquitous in “real‐world” clinical research (Wolpert & Rutter, 2018). For example, more than 40% of the data for duration of an untreated ED and more than 50% of EDE‐Q and CORE‐10/OM global score data were missing. There were many reasons for missingness including clinicians not being supported with or accustomed to data collection, difficulties obtaining data from patients, and limited capacity to collect, enter, and “chase” data (Richards et al., 2022). We were only able to include 3 out of 30 FREED services, as most only “came online” during the pandemic. Thus, only 502 of 2319 patients seen by all FREED services during the study period are represented here (21.6%). Additionally, site 3 only had 7 weeks of pre‐lockdown data. Despite these limitations, our findings allow some important conclusions to be drawn.

First, the significant increase in referrals is of concern. Even before the pandemic, English NHS services for EDs had been struggling to deliver timely well‐coordinated patient care, with notable failures in this area initiating a number of policy initiatives to reform and improve care. It is thus vital that these referral trends continue to be monitored carefully. There is some overlap between our findings and those seen in child and adolescent ED services in England. National data for child and adolescent ED services demonstrate a 1.2‐fold increase in referrals from 2019 to 2020 (7963 vs. 9758 referrals), while our FREED data shows a ~1.4‐fold increase in referrals for the same period (122 vs. 175 referrals), although this may be overestimated due to Site 3 joining in February 2020.

Second, our findings suggest that the increase in referrals was not driven by milder ED presentations. Instead, symptom severity stayed broadly stable over time. This contrasts with other studies with mixed or adult samples which reported greater symptom severity in ED presentations during the pandemic (e.g., Weissman & Hay, 2022). However, many such studies relied on retrospective recall (Linardon et al., 2022). Our study used clinical data collected at presentation to estimate symptom severity.

In this sample, there was an initial peak in the percentage of AN cases at onset of the first national lockdown and an upward shift until December 2020. These data align with the findings of a study comparing the full FREED‐4‐All data set to the earlier (pre‐pandemic) FREED‐Up study (Richards et al., 2022) and data from other countries, where increases in AN cases and related hospitalizations/presentations to ED services have been reported (Agostino et al., 2021; Springall et al., 2022). The combination of an increase in referrals and AN cases further supports the notion of increased pressures on services during the pandemic, as AN patients typically require multidisciplinary and more intensive treatment than other EDs.

Finally, mean duration of an untreated ED did not change significantly over the study period but did reflect heterogeneity, with a 17‐month difference between the monthly minimum and maximum points. Factors related to the COVID‐19 pandemic could have played some part in this heterogeneity; some young people may have sought treatment more quickly, either due to living at home with family because of lockdown restrictions, or becoming very unwell quickly (Weissman & Hay, 2022). In these cases, duration of an untreated ED would be shortened. Conversely, others may have delayed seeking care because of the publicized strain on health services, a fear of catching COVID‐19, and/or the longer waits if they sought help. The delayed increase in referrals (~6 months after the start of the first national lockdown) could be somewhat indicative of this delayed help seeking. It is also possible that the delayed increase is related to the potential influence of triggering/negative media content following the onset of the pandemic (Devoe et al., 2022). However, duration of an untreated ED remained relatively stable, which reflects the efforts by FREED services to seeing people quickly despite increasing referral numbers.

In summary, this is the first study to consider the specific impacts of COVID‐19 on presentations to care for young people with recent‐onset EDs accessing early intervention services. Although our work has limitations, findings point to persistent increases in referrals and AN diagnoses, at comparable levels of severity to pre‐pandemic times. A key factor in the success of implementing healthcare change is the ability of the innovation to adapt, respond, and work through changing contexts (Greenhalgh & Abimbola, 2019) and the continued provision of FREED through the pandemic suggests local teams are undertaking this adaptation. It is now important that evaluation continues to monitor longer‐term impacts of the pandemic, such as the impact on waiting times for assessment and treatment, and that investment matches the increased referral trends observed here.

## AUTHOR CONTRIBUTIONS

**Lucy Hyam:** Conceptualization; formal analysis; investigation; methodology; visualization; writing – original draft; writing – review and editing. **Katie Richards:** Conceptualization; formal analysis; investigation; methodology; visualization; writing – original draft; writing – review and editing. **Karina Allen:** Conceptualization; visualization; writing – original draft; writing – review and editing. **Ulrike Schmidt:** Conceptualization; visualization; writing – original draft; writing – review and editing.

## CONFLICT OF INTEREST

The authors declare no conflicts of interest.

## Supporting information


**APPENDIX S1** Supporting Information.

**APPENDIX S2** Supporting Information.

## Data Availability

The data set analyzed in this manuscript is not publicly available due to privacy and legal reasons declared in an operational agreement. This states that any data provided should not be shared with any external parties without express permission of the original owners of the data, and that these data may only be used for the purpose of evaluating the First Episode Rapid Early Intervention for Eating Disorders model.
