# Supplementary material for: The impact of the COVID‐19 pandemic on referral numbers, diagnostic mix, and symptom severity in Eating Disorder Early Intervention Services in England
Source: Int J Eat Disord. 2022 Oct 21;56(1):269–75. doi: 10.1002/eat.23836 (PMC9874422; doi:10.1002/eat.23836)
Supplement: Supplementary file 1 — APPENDIX S1 Supporting Information. [file EAT-56-269-s001.docx]

**Supplementary material 1 – Description of methods and run chart rules used.**

Run charts are line graphs that present a measure over time with the median plotted as a horizontal line splitting the data in two (Anhøj & Olesen, 2014). Probability based rules are used to objectively determine if the patterns in the data are random or non-random (Anhøj & Olesen, 2014; Perla, Provost & Murray, 2010). A run is a sequence of data points in a row on either side of the median line. In a process affected only by random variation, regular fluctuation above or below the median line is expected, but non-random variation indicates a pattern that is unstable or unpredictable, fluctuating too much or too little (Lloyd, 2017; Perla, Provost, & Murray, 2010). Perla, Provost & Murray (2010) provide a table of critical values to determine whether there are ‘too many’ or ‘too few’ runs in the data, and these rules can be applied to the data when there are ten or more data points available, which is appropriate for our dataset. Shifts in the data are indicated by six or more consecutive data points above or below the median line and allow us to determine non-random upwards or downward shifts in processes over time (Gauri, 2010; Lloyd, 2017).

**References**

Anhøj, J., & Olesen, A. V. (2014). Run charts revisited: a simulation study of run chart rules for detection of non-random variation in health care processes. *PLoS One*, *9*(11), e113825. https://doi.org/10.1371/journal.pone.0113825

Gauri, S. K. (2010). A quantitative approach for detection of unstable processes using a run chart. *Quality Technology & Quantitative Management, 7*(3), 231-247. https://doi.org/10.1080/16843703.2010.11673230

Lloyd, R. (2017). Quality health care: a guide to developing and using indicators. Jones & Bartlett Learning.

Perla, R. J., Provost, L. P., & Murray, S. K. (2011). The run chart: a simple analytical tool for learning from variation in healthcare processes. *BMJ quality & safety*, *20*(1), 46-51. http://dx.doi.org/10.1136/bmjqs.2009.037895
