# Supplementary material for: The impact of the COVID‐19 pandemic on referral numbers, diagnostic mix, and symptom severity in Eating Disorder Early Intervention Services in England
Source: Int J Eat Disord. 2022 Oct 21;56(1):269–75. doi: 10.1002/eat.23836 (PMC9874422; doi:10.1002/eat.23836)
Supplement: Supplementary file 2 — APPENDIX S2 Supporting Information. [file EAT-56-269-s002.docx]

**Supplementary material 2 – Run charts for monthly mean duration of an untreated eating disorder, BMI (AN patients), EDE-Q and CORE-10/OM**

*Figure 1*. Run charts depicting the mean monthly duration of an untreated eating disorder, and BMI (for AN patients) for FREED patients January 2019 – September 2021


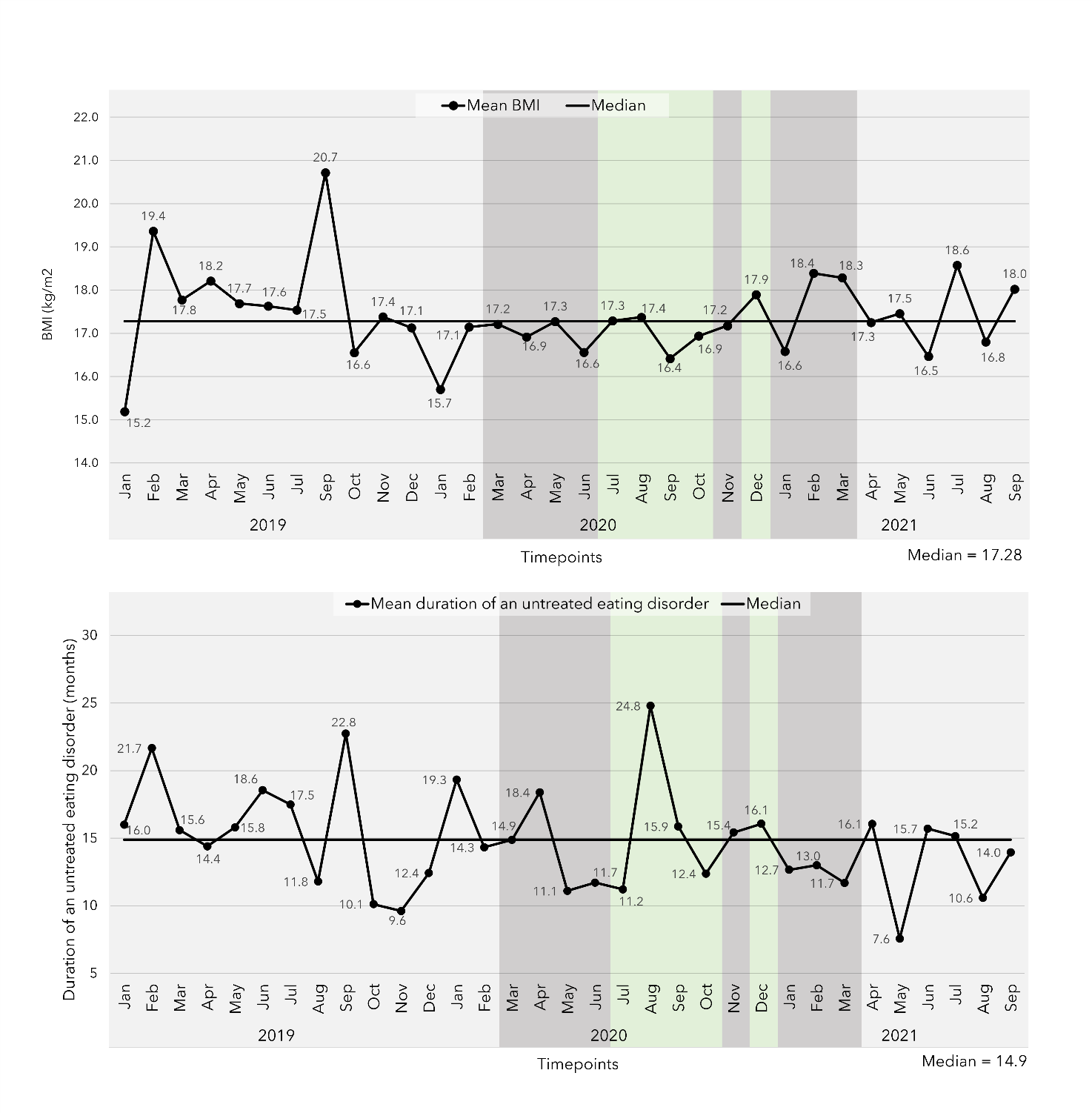


*Note.* The shaded areas of the charts represent the periods of different COVID-19 pandemic restrictions. Median values calculated for the monthly mean BMI/ duration of an untreated eating disorder, Jan 2019-Sept 2021.

*Figure 2.* Run charts depicting mean monthly CORE-10/OM and EDE-Q global scores for FREED patients January 2019 – September 2021


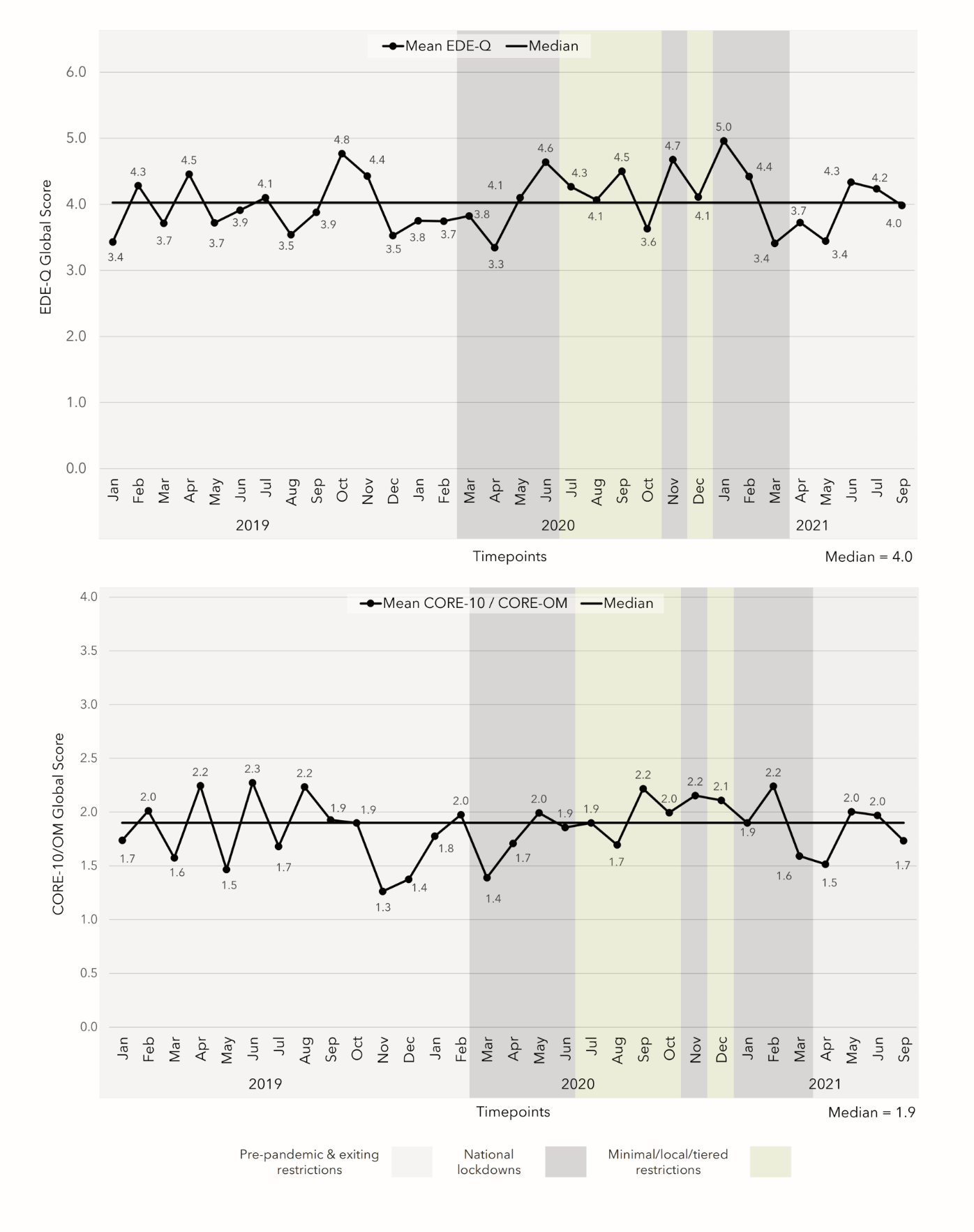


*Note.* The shaded areas of the charts represent the periods of different COVID-19 pandemic restrictions. Median values calculated for the monthly mean CORE-10/OM/EDE-Q Jan 2019-Sept 2021.
